# The significance of relative dose intensity in adjuvant chemotherapy of pancreatic ductal adenocarcinoma—including the analysis of clinicopathological factors influencing relative dose intensity

**DOI:** 10.1097/MD.0000000000004282

**Published:** 2016-07-22

**Authors:** Norimitsu Yabusaki, Tsutomu Fujii, Suguru Yamada, Kenta Murotani, Hiroyuki Sugimoto, Mitsuro Kanda, Goro Nakayama, Masahiko Koike, Michitaka Fujiwara, Yasuhiro Kodera

**Affiliations:** a Department of Gastroenterological Surgery (Surgery II), Nagoya University Graduate School of Medicine, 65 Tsurumai-cho, Showa-ku, Nagoya; b Center for Clinical Research, Aichi Medical University, 1–1 Yazakokarimata, Nagakute, Japan.

**Keywords:** adjuvant chemotherapy, PDAC, RDI

## Abstract

Recently, it has been reported that the relative dose intensity (RDI) of adjuvant chemotherapy (AC) influences survival in various cancers, but there are very few reports about RDI in pancreatic ductal adenocarcinoma (PDAC). The optimal timing for initiation of AC for PDAC also remains unknown. The aim of this study was to identify the significance of RDI and the time interval between surgery and initiation of AC on survival of patients with PDAC. Clinicopathological factors that affect RDI were also investigated.

A total of 311 consecutive PDAC patients who underwent curative resection between May 2005 and January 2015 were enrolled. Patients who underwent neoadjuvant chemoradiation, had UICC stage IV disease, or had early recurrences within 6 months were excluded, and the remaining 168 cases were analyzed.

Patients with RDIs ≥80% (n = 79) showed significantly better overall survival (OS) compared to patients with RDIs <80% (n = 55) (median survival time (MST): 45.6 months, 26.0 months, *P* < 0.001). Patients with no AC (n = 34) showed the worst OS (MST: 20.8 months). Whether the AC was initiated earlier or later than 8 weeks after surgery did not influence survival, either in patients with RDIs ≥80% (*P* = 0.79) or in those with <80% (*P* = 0.73). Patients in the S-1 monotherapy group (n = 49) showed significantly better OS than patients in the gemcitabine monotherapy group (n = 51) (MST: 95.0 months, 26.0 months, respectively; *P* = 0.001). Univariate analysis conducted after adjusting for the chemotherapeutic drug used identified several prognostic factors; male gender (*P* = 0.01), intraoperative blood transfusion (*P* = 0.005), lymph node metastasis (*P* = 0.03), and postoperative WBC count (*P* = 0.03). Multivariate analysis identified intra-plus postoperative blood transfusion (*P* = 0.002) and high postoperative platelet-to-lymphocyte ratios (PLR) (*P* = 0.04) as independent predictors of poor RDI.

Efforts to maintain RDI had a greater impact on survival than the struggle to start AC early after surgery. Intra-plus postoperative blood transfusion and a high postoperative PLR could be predictive markers of reduced RDI in AC of PDAC patients. Avoidance of perioperative blood transfusions where possible and nutritional support during the perioperative period could maintain adequate RDI and may lead to improved long-term outcome.

## Abstract 2

Supplemental Digital Content is available in the text

## 1 Introduction

The prognosis of pancreatic ductal adenocarcinoma (PDAC) remains poor despite recent improvements in surgical technique,^[1]^ postoperative management,^[2]^ and neoadjuvant and adjuvant chemotherapy (AC).^[3]^ The European Study Group for Pancreatic Cancer (ESPAC) demonstrated through several large-scale randomized controlled studies that AC should be the standard of care for resectable PDAC;^[4–7]^ these studies demonstrated that gemcitabine or fluorouracil plus folic acid significantly improved the prognosis of PDAC compared to surgery alone. In Japan, following the results of the Japan Adjuvant Study Group of Pancreatic Cancer (JASPAC)-01,^[8]^ adjuvant S-1 chemotherapy was established as a standard of care for resectable PDAC.

In several cancers treated with AC, a decreased relative dose intensity (RDI) has been associated with a poor prognosis.^[9–13]^ In PDAC patients, however, there are only two reports concerning RDI of AC and prognosis^[14,15]^ and the factors affecting RDI. Furthermore, starting AC within less than 8 weeks has been recommended in colon cancer management,^[16,17]^ although the optimal timing for initiation of AC for PDAC remains unknown.

The aim of this study was to investigate the effect of the RDI and the time interval between surgery and initiation of AC on survival of patients with PDAC. Clinicopathological factors affecting the RDI of AC were also explored.

## 2 Methods

### 2.1 Patients

We enrolled 311 consecutive PDAC patients who planned to undergo curative resection at the Department of Gastroenterological Surgery, Nagoya University Hospital, Japan, between May 2005 and January 2015. The histological diagnosis of PDAC was confirmed for all patients. Written informed consent for inclusion in the study, as required by the Institutional Review Board of Nagoya University, was obtained from all patients.

Of these 311 patients, 177 patients were excluded for the following reasons: received neoadjuvant chemoradiation (n = 41), diagnosed as having distant metastasis during surgery (n = 21), underwent middle pancreatectomy due to different preoperative diagnosis (n = 1), surgical death (n = 2), followed by another hospital after discharge (n = 59), and curative resection without adjuvant chemotherapy (AC) (n = 34). As a result, 153 patients received AC after surgery at our institution (Supplemental Fig. 1). In these patients, 19 developed early recurrence within 6 months.

### 2.2 Surgical procedures

All PDACs were determined to be surgically resectable before surgery. Operative methods were determined based on tumor location. All patients underwent regional lymph node dissection. If portal vein infiltration was suspected during surgery, combined portal vein resection was performed. Reconstruction after pancreatoduodenectomy (PD) was performed according to the modified Child method.^[18]^ The pathological stage of PDAC was assessed according to the TNM classification system of malignant tumors published by the International Union Against Cancer [Union for International Cancer Control (UICC), 7th edition].

### 2.3 Chemotherapy regimens

The AC regimen used for each patient was either S-1 monotherapy (n = 49) or gemcitabine monotherapy (n = 51). Gemcitabine had been the drug of choice for AC until the result of JASPAC-01^[8]^ was disclosed in our country. After the disclosure, essentially all the patients with PDAC were administered S-1. The gemcitabine plus S-1 regimen (n = 34) was only used for patients registered in clinical trials. The S-1 regimen consisted of 80 to 120 mg, depending on body surface area, administered twice a day for 4 weeks and repeated every 6 weeks for 4 courses, or for 2 weeks and repeated every 3 weeks for 8 cycles. The gemcitabine regimen consisted of 1000 mg/m^2^ given by intravenous infusion over a 30-minute period. Patients receiving gemcitabine were scheduled for 6 cycles every 4 weeks followed by a 1-week pause. In the gemcitabine plus S-1 regimen, patients received gemcitabine (800 mg/ m^2^, day 1) plus S-1 (65 mg/ m^2^, days 1–7) every 2 weeks for 12 cycles.

AC was planned to be 6 months in duration and was initiated as soon as possible after the patient was discharged from the hospital. Postoperative blood test data were evaluated at the onset of AC, and both blood tests and clinical symptoms were checked every visit. Computed tomography was performed every 3 months after discharge to monitor relapse.

### 2.4 Relative dose intensity (RDI)

RDI was calculated for each patient according to the method proposed by Hryniuk and Bush.^[19]^ For each drug within each regimen, the total dosage that the patient received was divided by the total dosage specified by the corresponding standard regimen. Since the median value of the RDI was 80%, we set a cutoff value of 80% and defined an RDI ≥ 80% as complete AC and an RDI <80% as incomplete AC.

### 2.5 Postoperative complications and definitions

Postoperative complications were evaluated by means of a modified Clavien grading system. A complication of grade III or higher was considered a clinically significant event. The classification system of the International Study Group on Pancreatic Fistula (ISGPF) was used to predict postoperative pancreatic fistula.

### 2.6 Statistical analysis

Continuous variables are expressed as the mean ± standard deviation and the range. Overall survival (OS) was calculated using the Kaplan–Meier method, and the differences in survival curves were analyzed using the log-rank test. Univariate and multivariate logistic regression analyses were performed to analyze factors associated with complete AC. In addition, multivariate Cox proportional hazard model was used to determine independent risk factors associated with OS. Data were analyzed using JMP v10 software (JMP, SAS Institute, Cary, NC). The level of statistical significance was defined as *P* < 0.05.

## 3 Results

### 3.1 Patient characteristics

Patient demographics and clinical characteristics are listed in Table 1. Among 134 patients, 79 (61 men and 18 female) had RDIs ≥80%, and 55 (25 men and 30 female) had RDIs <80%. The median follow-up period was 24.5 months (range 3.8–95.0 months). Eighty-eight (65.7%) patients developed recurrence, and 64 (47.8%) patients died before the end of the follow-up period. The major reasons for reduction of the RDI included neutropenia (n = 21), physician's judgment (n = 15), general fatigue (n = 10), gastrointestinal toxicity (n = 8), and refusal to continue with the chemotherapy (n = 5). Physician's judgment denotes either dose reduction, alteration of the treatment schedule or both, not through observation of definite criteria such as the laboratory data but based on vulnerability of the patient as judged from the age, physical status and complaints. Refusal to continue chemotherapy means patient refusal due to various reasons other than unacceptable toxicities, such excessive concerns about the antineoplastic agents aroused by what the medical staff considered as mild toxicity and/or various and often erroneous information against these agents which are readily accessible through the internet and other sources. RDIs <80% were particularly common in women (*P* < 0.001). Patients with RDIs <80% also had significantly more combined portal vein resections (*P* = 0.02), intra-plus postoperative blood transfusions (*P* = 0.007), lymph node metastases (*P* = 0.001), dissected peripancreatic tissue margins (pDPM) (+) (*P* = 0.004), lower postoperative serum albumin levels (*P* < 0.001), lower white blood cell (WBC) counts (*P* = 0.03), and higher platelet-to-lymphocyte ratios (PLR) (*P* = 0.02). The postoperative blood transfusion was defined as transfusion during hospitalization. A total of 18 patients received intra-plus postoperative blood transfusion, and the breakdowns were as follows; both intra and postoperative (n = 2), intraoperative (n = 13), and postoperative (n = 3). In addition, there were 34 patients who did not receive AC. These patients were significantly older than the patients with RDIs<80% (73.7 ± 7.6, 64.4 ± 8.6 years, *P* < 0.001) and included 3 patients who were under dialysis, but there were no significant differences regarding other clinicopathological factors.

**Table 1:**
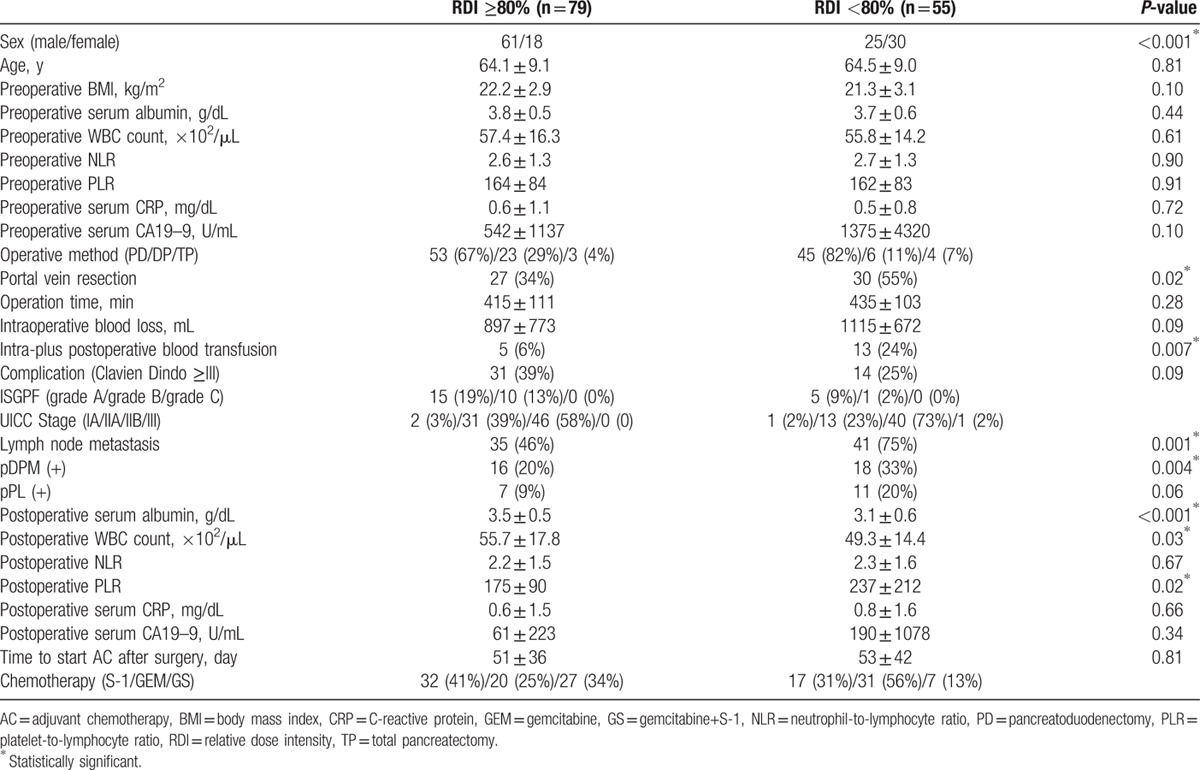
Patient characteristics.

### 3.2 Overall survival

**Figure 1:**
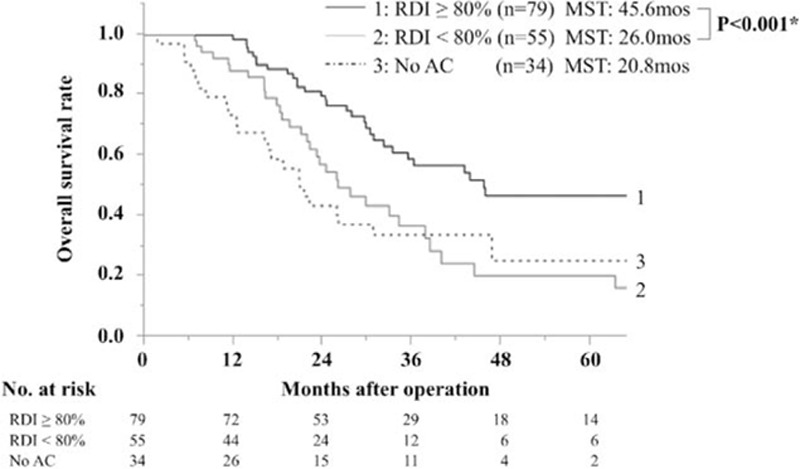
OS of patients according to the RDI of AC. Patients with an RDI ≥80% showed significantly better OS than patients with an RDI <80% (≥80%; MST: 45.6 months, <80%; 26.0 months; *P* < 0.001). Patients with no AC showed the worst OS (MST: 20.8 months). AC = adjuvant chemotherapy, MST = median survival time, OS = overall survival, RDI = relative dose intensity.

**Figure 2:**
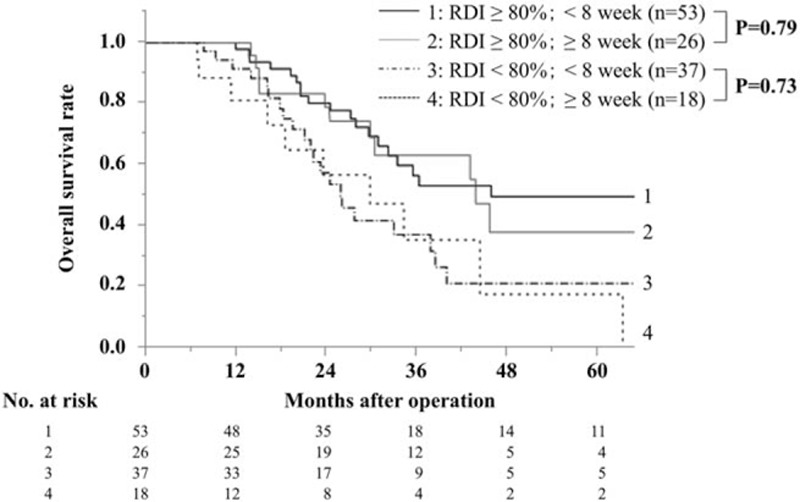
OS of patients according to the RDI and AC start time. In the RDI ≥80% group, no significant difference was found in OS according to the AC start time (MST: 45.8 months, 43.8 months; *P* = 0.79). In addition, no significant difference was found in the RDI <80% group (MST: 25.8 months, 29.7 months, *P* = 0.73). AC = adjuvant chemotherapy, MST = median survival time, OS = overall survival, RDI = relative dose intensity.

The OS of patients stratified by the RDI is shown in Fig. 1. Patients with RDIs ≥80% showed significantly better OS than patients with RDIs <80% [≥80%; median survival time (MST) 45.6 months, <80%; 26.0 months, *P* < 0.001]. Patients with no AC showed the worst OS (MST: 20.8 months). Patients were then classified into 4 groups according to both the RDI and the time from surgery to AC as follows: Group 1, RDI ≥80%/ <8 weeks (n = 53); Group 2, RDI ≥80%/ ≥8 weeks (n = 26); Group 3, RDI <80%/ <8 weeks (n = 37); and Group 4, RDI <80%/ ≥8 weeks (n = 18) (Fig. 2). In the group of patients with an RDI ≥80%, a delay in the initiation of AC did not significantly affect the OS (*P* = 0.79), and the MST was 45.8 months in Group 1 and 43.8 months in Group 2. Similarly, no effect was observed in the group with an RDI of less than 80% (*P* = 0.73); the MST was 25.8 months in Group 3 and 29.7 months in Group 4. In the group of patients who started AC within 8 weeks, the RDI significantly affected the OS (Groups 1 and 3, *P* = 0.005). Similarly, a trend was observed for RDI to correlate with OS in the group starting AC after the first 8 weeks (Groups 2 and 4, *P* = 0.06).

We also analyzed OS according to the chemotherapy regimen. In the S-1 monotherapy group, patients with RDIs ≥80% (n = 32) showed significantly better OS than patients with RDIs <80% (n = 17) (Supplemental Fig. 2) (5-year survival rate: 67.4%, 31.4%, respectively; *P* = 0.02). In the gemcitabine monotherapy group, there was also a significant difference in OS between patients with RDIs ≥80% (n = 20) and patients with RDIs <80% (n = 31) (Supplemental Fig. 2) (MST: 32.1 months, 23.1 months, respectively; *P* = 0.03). The patients in the S-1 monotherapy group showed significantly better OS than patients in the gemcitabine monotherapy group (MST: 95.0 months, 26.0 months, respectively; *P* = 0.001). There were significantly more patients with RDIs ≥80% in the S-1 monotherapy group than in the gemcitabine group (65%, 39%, *P* = 0.009; respectively) (Supplemental Table 1), and there were significantly more patients with advanced pathological UICC stages in the gemcitabine monotherapy group than in the S-1 monotherapy group (*P* = 0.002) (Supplemental Table 2). For these reasons, the following analyses were conducted to identify predictive factors for poor RDI after adjusting for the anticancer drug used during AC.

### 3.3 Predictive factors for completing AC

Univariate analysis was performed using various preoperative, operative, and postoperative factors and including the drug used for AC (either S-1 (n = 49) or gemcitabine (n = 51)) as covariates (Table 2). This analysis identified 4 prognostic factors: male gender [odds ratio (OR) 3.0, 95% confidence interval (CI): 1.3–7.3, *P* = 0.01], intra-plus postoperative blood transfusion (OR: 0.1, CI: 0.03–0.5, *P* = 0.003), lymph node metastasis (OR: 0.4, CI: 0.2–0.9, *P* = 0.03), and postoperative WBC count (OR: 1.03, CI: 1.00–1.06, *P* = 0.03). We performed a multivariate analysis using 9 factors that showed a meaningful tendency in the univariate analysis (Table 3). Multivariate analysis showed a similar trend, with male gender (OR: 3.8, CI: 1.4–11.3, *P* = 0.01), intra-plus postoperative blood transfusion (OR: 0.1, CI: 0.02–0.5, *P* = 0.002), and postoperative PLR (OR: 0.99, CI: 0.99–0.99, *P* = 0.04) identified as independent predictors for completion of AC. We also performed univariate and multivariate analyses of clinicopathological factors for OS (Supplemental Table 1). Univariate analysis of OS using the Cox proportional hazards model identified the poor prognostic factors as follows: lymph node metastasis, incomplete AC, and low postoperative serum albumin level. The results of multivariate analysis revealed that complete AC was independently associated with good prognosis (hazard ratio = 0.5; 95% confidence interval, 0.2–0.9; *P* *=* 0.03).

**Table 2:**
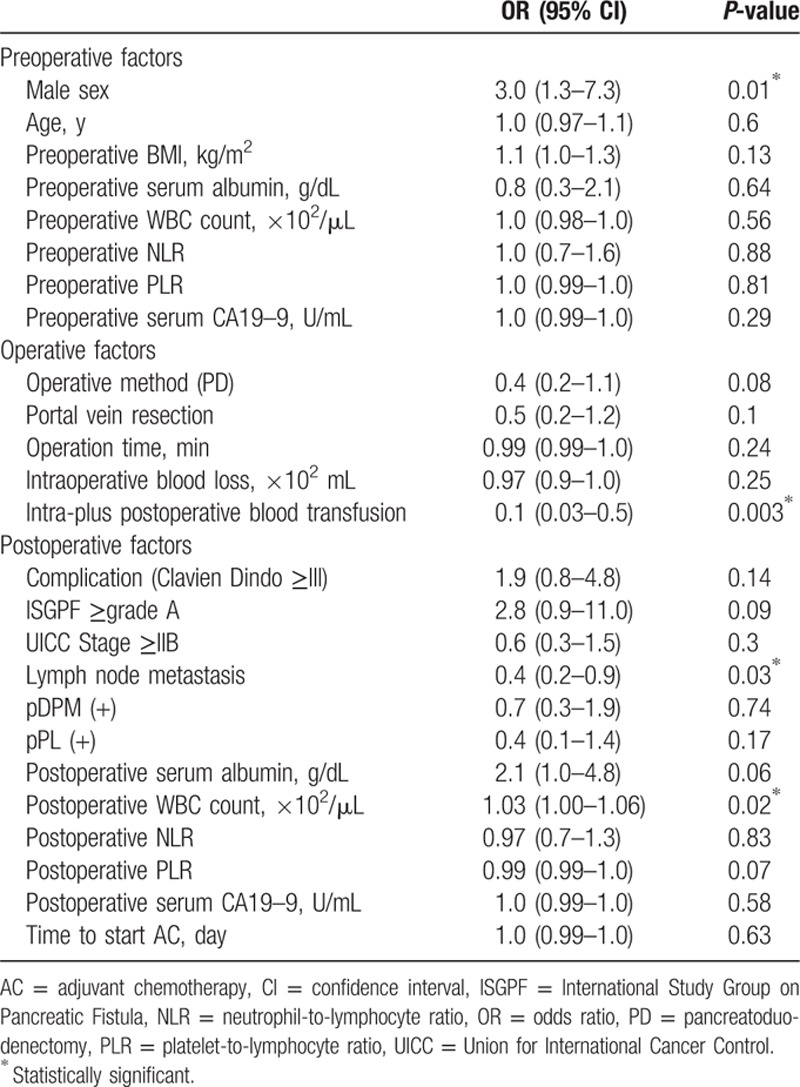
Univariate logistic regression analysis of factors related to completing AC, adjusted for chemotherapy regimen.

**Table 3:**
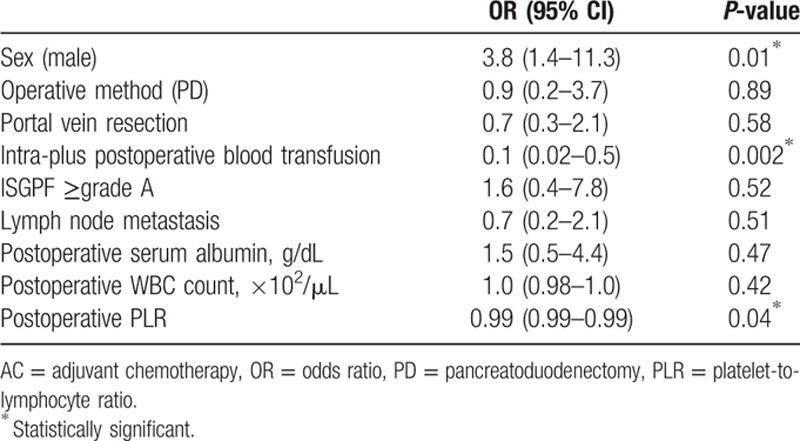
Multivariate logistic regression analysis of various factors related to completing AC adjusted for chemotherapy regimen.

## 4 Discussion

Use of a sufficient dosage and drug administration according to the schedule determined by clinical trials are the basic principles of chemotherapy for cancer. Thus, the RDI is often considered an important prognostic factor among patients who undergo postoperative adjuvant chemotherapy. To our knowledge, however, only two reports have been published concerning the RDI of AC for patients with PDAC.^[14,15]^ In these reports, high RDIs and successful execution of the planned treatment were associated with an improved prognosis. The results of the present study were consistent with these reports, as a decrease in the RDI was strongly associated with a poor prognosis. In addition, the postoperative PLR, one of the nutritional indexes that predict survival, also affected the RDI. This was also consistent with the earlier report that the postoperative serum albumin level was an independent predictor for maintaining the RDI. However, unlike the case of colorectal cancer, a time interval of longer than 8 weeks before initiation of AC did not adversely influence survival.

Postoperative nutritional status in terms of the loss in body weight and lean body mass is considered to affect adherence to postoperative chemotherapy in patients with gastric cancer.^[20]^

Surgery for PDAC is well-recognized as one of the most invasive procedures among abdominal surgeries and is associated with high morbidity, a decrease in food intake and deficiency in the absorption of nutrients. Therefore, the nutritional status of patients with PDAC is also aggravated after surgery, possibly resulting in poor adherence to AC, especially when attempts are made to initiate it early after surgery. Moreover, several scoring systems that reflect nutritional status, such as the prognostic nutritional index (PNI), neutrophil lymphocyte ratio (NLR), and Glasgow prognostic score (GPS), have been associated with a poor prognosis in PDAC patients.^[21,22]^ Because the PNI and GPS greatly depend on the serum albumin level, we substituted the albumin level for these measures. In the current study, the PLR was shown to be a significant predictor of the RDI. Platelets and lymphocytes are considered to be significant parameters related to the immune condition, and the lymphocyte count in particular reflects ability of the individual to eliminate tumor cells.^[23]^ Actually, there are some reports to demonstrate that high lymphocytic infiltration to the tumor site could be associated with superior response to systemic chemotherapy.^[24,25]^ In PDAC, the PLR was reported to correlate with prognosis after surgery.^[26]^ To the best of our knowledge, there have been no reports implicating correlation between toxicity of anticancer agent and PLR. However, high lymphocytes might be associated with reduced toxicity through enhanced immune status and increased sensitivity to the chemotherapy.

The fact that early enteral feeding (EEN) after a pancreaticoduodenectomy (PD) was shown to be more useful in maintaining body weight and recovering digestive function than total parenteral nutrition (TPN) may offer clues for counteracting this situation.^[27]^ Further clinical trials may be warranted to confirm that such nutritional support actually translates into improvement in survival.

In contrast, the postoperative serum albumin levels of patients who received a PD were significantly lower than those of patients who underwent other operative methods (*P* < 0.001, data not shown). The RDI of patients who received a PD tended to be lower than those of patients who underwent other methods (*P* = 0.06, data not shown). Although the operative method was not a meaningful factor in the multivariate analysis in this study, more rigorous perioperative nutritional support might be necessary, specifically for patients who receive a PD.

Additionally, patients who received an intra-plus postoperative blood transfusion tended to have lower RDIs. Previously, some studies have shown that patients with PDAC who receive a perioperative blood transfusion show a poorer prognosis.^[28–30]^ In addition, intraoperative blood transfusions were shown to be an independent prognostic factor in PDAC.^[31]^ Although the underlying mechanism remains unclear, blood transfusions have an immunosuppressive effect via transfusion-related immune modulation.^[32]^ It was also proposed that following blood transfusion, lipid mediators,^[33]^ pro-inflammatory cytokines,^[34]^ and immunosuppressive proteins^[35]^ in the transferred blood might suppress natural killer cell activity and IL-2 production.^[36]^ This immunosuppression at the cellular level might influence tolerability of the chemotherapy. As shown in Table 1, the patients with RDIs <80% had significantly more lymph node metastasis, portal vein resection, and pDPM(+) than those with RDIs ≥80%. Therefore, it is certain that patients with RDIs <80% received more invasive surgery for more advanced cancer than those with RDIs ≥80%. In addition, immunosuppression by the blood transfusion might result in greater susceptibility to toxicity due to the AC. In this regard, we think that it is important to avoid unnecessary blood transfusion and try to reduce intraoperative bleeding where possible.

There were several limitations of our study. First, this was a retrospective single-institution study with a limited number of patients. Second, the anticancer agent used in the adjuvant therapy was varied and was selected based on the physician's preferences. The outcomes of patients receiving S-1 monotherapy were actually significantly better than those of patients receiving gemcitabine, as has been shown by the JASPAC-01 trial.^[8]^ Although analyses to determine predictive factors for the RDI were performed after adjusting for the drugs used, our data might still suffer from bias due to drug choice. Third, no standard protocol for blood transfusions was used; the decision to transfuse was based on the judgment of the anesthesiologist.

In conclusion, the maintenance of RDI is important to improve prognosis. Intra-plus postoperative blood transfusion and a high postoperative PLR could be predictive markers of reduced RDI in AC of PDAC patients. In addition, maintaining RDI is possibly more important to patient outcome than starting AC early after surgery. The role of rigorous perioperative nutritional support may be worth exploring in future studies.

## Supplementary Material

Supplemental Digital Content
