# Supplementary material for: The significance of relative dose intensity in adjuvant chemotherapy of pancreatic ductal adenocarcinoma—including the analysis of clinicopathological factors influencing relative dose intensity
Source: Medicine (Baltimore). 2016 Jul 22;95(29):e4282. doi: 10.1097/MD.0000000000004282 (PMC5265784; doi:10.1097/MD.0000000000004282)
Supplement: Supplemental Digital Content [file medi-95-e4282-s001.doc]

Supplementary Figure 1. Study flow diagram.


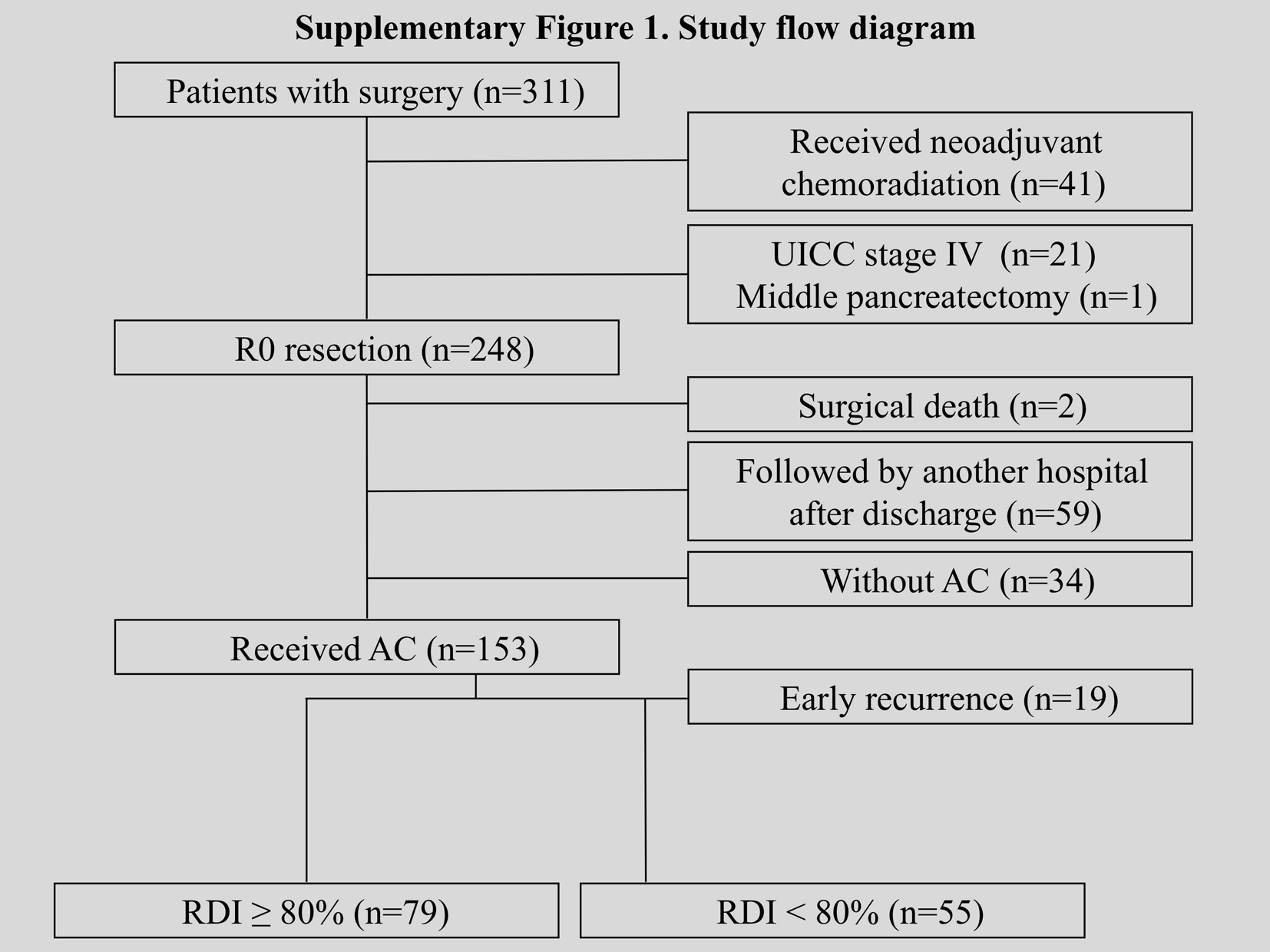


AC: adjuvant chemotherapy

Supplementary Figure 2. OS according to RDI and chemotherapy regimen.


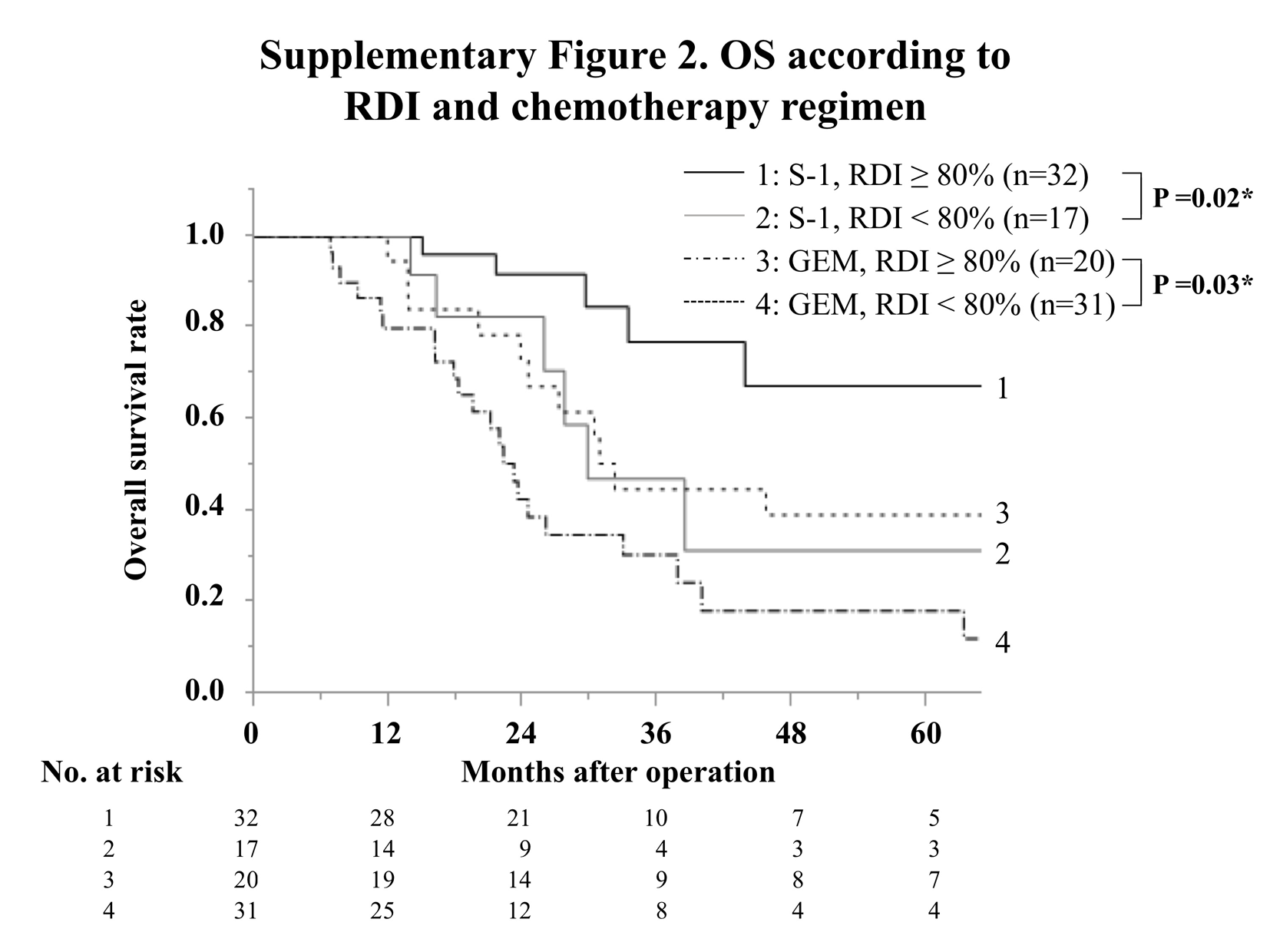


In both the S-1 and GEM monotherapy groups, patients with RDIs ≥80% showed significantly better OS than patients with RDIs <80% (P = 0.02, 0.03).

**Supplementary Table 1. Univariate and multivariate analyses of clinicopathological factors for**

**OS adjusted for chemotherapy regimen**

| **Variables** | **Univariate analysis** | | |  | **Multivariate analysis** | | |
| --- | --- | --- | --- | --- | --- | --- | --- |
|  | **HR** | **95% CI** | **P-value** |  | **HR** | **95% CI** | **P-value** |
| **Sex (male)** | 0.8 | 0.5-1.5 | 0.55 |  |  |  |  |
| **Age (years)** | 0.98 | 0.95-1.0 | 0.28 |  |  |  |  |
| **Portal vein resection** | 1.3 | 0.7-2.3 | 0.46 |  |  |  |  |
| **Intraoperative blood loss (×10^2^)** | 1.0 | 0.98-1.1 | 0.28 |  |  |  |  |
| **Intra-plus postoperative blood transfusion** | 1.6 | 0.6-3.3 | 0.31 |  |  |  |  |
| **Complication (Clavien Dindo ≥III)** | 0.7 | 0.3-1.2 | 0.19 |  |  |  |  |
| **UICC T3,T4** | 0.96 | 0.2-17.7 | 0.97 |  |  |  |  |
| **Lymph node matastasis** | 2.4 | 1.2-4.9 | 0.009* |  | 1.6 | 0.7-3.7 | 0.23 |
| **pPL(+)** | 2.1 | 0.9-4.3 | 0.07 |  | 1.5 | 0.6-3.1 | 0.35 |
| **pDPM(+)** | 1.4 | 0.7-2.6 | 0.3 |  |  |  |  |
| **RDI ≥80%** | 0.4 | 0.2-0.7 | 0.003* |  | 0.5 | 0.2-0.9 | 0.03* |
| **Time to start AC <8 week** | 0.9 | 0.5-1.8 | 0.8 |  |  |  |  |
| **Postoperative PLR** | 1.0 | 0.99-1.0 | 0.51 |  |  |  |  |
| **Postoperative serum–albumin (g/dl)** | 0.6 | 0.3-0.9 | 0.04* |  | 0.8 | 0.5-1.5 | 0.56 |

* statistically significant

*HR* hazard ratio
